# Large giraffids (Mammalia, Ruminantia) from the new late Miocene fossiliferous locality of Kemiklitepe-E (Western Anatolia, Turkey)

**DOI:** 10.1007/s12549-020-00433-4

**Published:** 2020-09-02

**Authors:** Alexandros Xafis, Serdar Mayda, Mehmet Cihat Alçiçek, Tanju Kaya, Kazım Halaçlar, Friðgeir Grímsson, Doris Nagel

**Affiliations:** 1 Department of Palaeontology, Faculty of Earth Sciences, University of Vienna, 1090 Vienna, Austria; 2 Department of Biology, Faculty of Science, Ege University, 35100 Izmir, Turkey; 3 Department of Geology, Pamukkale University, 20070 Denizli, Turkey; 4 Natural History Museum, Ege University, 35100 Izmir, Turkey; 5 Institute of Vertebrate Paleontology and Paleoanthropology, Chinese Academy of Sciences, Beijing, 100044 China; 6 Department of Botany and Biodiversity Research, University of Vienna, 1030 Vienna, Austria

**Keywords:** Artiodactyla, Giraffidae, *Helladotherium*, *Samotherium*, Turolian, MN12

## Abstract

Kemiklitepe is a well-known locality with four recognised fossiliferous horizons, KTA to KTD, which have yielded a plethora of mammalian remains. Previous taxonomic studies indicate the presence of three giraffid taxa: *Samotherium major* and *Palaeotragus rouenii* from the uppermost three horizons, KTA, KTB and KTC, as well as *Palaeotragus rouenii* and *Samotherium*? sp. from the lowermost KTD horizon. In this study a new locality, Kemiklitepe-E, is presented for the first time. Kemiklitepe-E is located approximately 350 m NW of the classic Kemiklitepe locality. The fossiliferous sedimentary rocks at Kemiklitepe-E occur at the same stratigraphic level as localities KTA, KTB and KTC. The preliminary faunal list includes representatives of Proboscidea, Chalicotheriidae, Equidae, Bovidae and Giraffidae. Comprehensive descriptions and comparisons of the Kemiklitepe-E Giraffidae specimens suggest the co-occurrence of two large giraffids: *Samotherium major* and *Helladotherium duvernoyi*. *Samotherium major*, previously documented from this region, is the most common taxon at Kemiklitepe. *Helladotherium duvernoyi* is rare at Kemiklitepe and here reported for the first time. The two taxa coexisted during the middle Turolian in Greece and Western Anatolia. In addition, it is suggested that specimens of *Samotherium*? sp described from KTD possibly belong to *Samotherium neumayri*. Based on the stratigraphic position of fossiliferous rocks, as well as the faunal data presented herein, the newly discovered locality is considered to be of middle Turolian (MN12) age.

## Introduction

The family Giraffidae includes pecoran ruminants, which, together with the Palaeomerycidae, are part of a large clade called Giraffomorpha (Sánchez et al. 2015). Giraffids, which most likely originated during the late Oligocene (Mennecart et al. 2019) are characterised by the presence of ossicones, which are epiphyseal cranial appendages, bilobed canines and long limb bones (Janis and Scott 1987; Solounias 1988, 2007; Harris et al. 2010; Grossman and Solounias 2014).

Representatives of Miocene Giraffidae are recurrent components of the so-called “Pikermian Biome”, which used to extend from the Iberian Peninsula to East Asia (Danowitz et al. 2016 and literature cited therein). Common late Miocene large-sized giraffid taxa are *Helladotherium* and *Samotherium*. *Helladotherium* is monospecific and *H. duvernoyi* Gaudry and Lartet (1856) constitutes one of the most common large-sized Miocene Giraffidae of the Eastern Mediterranean. *Samotherium* was a widespread genus and its largest species, *S. major* Bohlin (1926), is a common element of late Miocene fossiliferous sites but has only been reported from few Greek and Anatolian localities.

Kemiklitepe-E is a newly discovered fossiliferous outcrop in Western Anatolia. All vertebrate remains were originally excavated and collected by local authorities and inhabitants, as well as private collectors during the early 2000s. The collected material was subsequently transported to the Ege University Natural History Museum (EUNHM) where it is currently stored and partly exhibited.

This study presents the first description of the new fossiliferous locality, and includes a preliminary stratigraphic outline, as well as the first information on the fossil fauna. The main focus of this report is on dental and postcranial material of Giraffidae. The primary objective is to enrich our knowledge on the late Miocene giraffids from Anatolia, and to provide the first chronostratigraphic framework for Kemiklitepe-E.

The stratigraphy and fauna at Kemiklitepe-E are currently under study. However, preliminary faunal list reveals a typical Turolian assemblage composed of *Choerolophodon pentelici* Gaudry and Lartet (1856), *Hiparrion* spp., *Ancylotherium pentelicum* Gaudry and Lartet (1856), *Palaeoryx* sp., *Tragoportax* sp., and *Gazella* sp.

## Geological setting and previous work

**Fig. 1:**
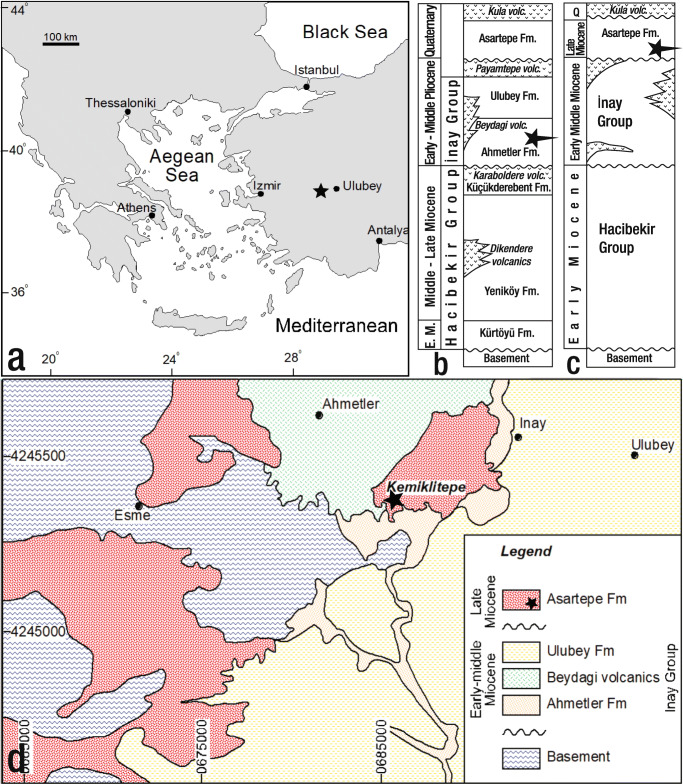
**a** Regional location of Kemiklitepe-E locality. **b** Stratigraphic position of the Kemiklitepe fossil locality proposed by Şen et al. (1994) (modified by Ercan et al. 1978). **c** Stratigraphic position of the Kemiklitepe fossil locality revised by Seyitoğlu et al. (2009). **d** Geological map of the Kemiklitepe fossil locality and its surroundings (based on Karaoğlu et al. 2010). Asterisks indicate the position of Kemiklitepe fossil locality

The fossiliferous locality at Kemiklitepe (N 38° 23′ 50.1′′, E 29° 08′ 54.2′′) is located in Western Anatolia, approximately 15 km west of the town Eşme, in the Uşak Province, Western Turkey (Sen et al. 1994; Saraç 2003; Fig. 1a–d). The first fossils from Kemiklitepe were studied and described by Yalçınlar (1946). Subsequent research on fossil remains was conducted by Ozansoy (1957, 1969), Becker-Platen et al. (1975), Tuna (1985) and Şen et al. (1994). The latter was a part of an extensive monograph on the stratigraphy and palaeoecology of the site, including a comprehensive description of all mammal remains. Şen et al. (1994) suggested an age of ~ 7.7–7.1 Ma for the sediments at Kemiklitepe based on magnetostratigraphic correlation, which was also supported by the typically Turolian fossil mammal assemblage. According to geological maps from the Geological Survey of Turkey (MTA), the fossiliferous sediments at Kemiklitepe are part of the Ahmetler Fm (Balçıklıdere Mbr) and overlain by the Ulubey Fm (Ercan et al. 1978, 1980, 1983; Fig. 1b). However, recent field investigations by Seyitoğlu et al. (2009) and Karaoğlu et al. (2010) clearly show that these fossiliferous sediments belong to the Asartepe Fm, and are situated on top of the Ulubey Fm (Fig. 1c). The Asartepe Fm is composed of massive mudstones with matrix supported fine-grained conglomerate alternations representing subaerial deposition in a distal alluvial-fan environment. The conglomerates consist of pebbles to boulders derived from the underlying metamorphic basement rocks of the lacustrine Ulubey Fm and the volcanic Beydağı Fm (e.g. Seyitoğlu et al. 2009). The fossiliferous sediments of the Asartepe Fm were deposited following an erosional stage (see fig. 7 in Seyitoğlu et al. 2009), as can be observed at the valley floor where the contact zone between the Ulubey Fm and the Asartepe Fm can be traced (e.g. Ercan et al. 1978; Seyitoğlu et al. 2009).

Previously recognised fossiliferous horizons at Kemiklitepe are the upper and younger sites KTA, KTB and KTC, as well as the stratigraphically lower KTD (Sen et al. 1994). Faunal and magnetostratigraphic correlations suggest a woodland/forest environment with an age of ~ 7.1 Ma for KTA, KTB and KTC, and ~ 7.6 Ma for KTD (Şen et al. 1994). The newly discovered locality, Kemiklitepe-E (N 38° 23′ 58′′, E 29° 08′ 47′′), is found approximately 350 m NW of the classic Kemiklitepe locality. The fossiliferous sediments at Kemiklitepe-E occur at the same stratigraphic level as horizons KTA, KTB and KTC found at the historic outcrop. Previous taxonomic work has revealed the presence of possibly three giraffid taxa: *Samotherium major* and *Palaeotragus rouenii* Gaudry (1861) from the upper horizons (KTA, KTB, KTC) and *Samotherium*? sp. and *P. rouenii* from the lower horizon (KTD). Kemiklitepe-E has yielded a small collection of two large Giraffidae, which are described herein, while small or intermediate-sized giraffids are absent.

## Materials and methods

The fossil material from Kemiklitepe-E is stored in the collection of the Natural History Museum of Ege University, Izmir, Turkey (EUNHM). All measurements of dental and postcranial material were taken with an electronic calliper, with a precision of ± 0.2 mm, and are given in millimetres (Table 1; Table 2). For dental elements, width measurements were taken at the base of the crown, and length measurements represent the maximum anteroposterior measure. All statistical analyses and graphs were compiled using PAST (Hammer et al. 2001). The terminology follows Bärmann and Rössner (2011) for dental material, Solounias and Danowitz (2016a) for the astragali, Ríos et al. (2016) for the metapodials and Schaller (2007) for the remaining postcranial elements.

**Table 1:** Measurements of post-cranial elements of *Helladotherium duvernoyi* from Kemiklitepe-E.

**Element**	**Inventory number**	**Sin/Dex**	**Lmax**	**Lmin**	**TDmax**	**APDmax**	**TDtuber**	**APDtuber**
Calcaneus	UEK-E/PV-4532	dex	196.10	130.35	62.39	-	54.13	66.08
Calcaneus	UEK-E/PV-4533	sin	-	133.06	-	-	57.82	67.58
**Element**	**Inventory number**	**Sin/Dex**	**Llat**	**Lmed**	**TDprox**	**TDdis**		
Astragalus	UEK-E/PV-4534	dex	103.82	92.97	74.51	77.51		
Astragalus	UEK-E/PV-4536	dex	-	90.08	-	-		
**Element**	**Inventory number**	**L**	**TDprox**	**APDprox**	**TDdis**	**APDdis**		
Proximal phalanx	UEK-E/PV-4541	102.69	49.14	-	44.96	31.22		
Proximal phalanx	UEK-E/PV-4542	101.45	49.90	-	44.85	34.39		
Middle phalanx	UEK-E/PV-4544	60.45	45.98	48.78	43.89	42.19		
Middle phalanx	UEK-E/PV-4546	-	44.11	44.09	-	-		

*Sin*, left; *Dex*, right; *TD*, transverse diameter; *APD*, antero-posterior diameter; *L*, length; *prox*, proximal; *dia*, diaphysis; *dis*, distal; *lat*, lateral; *med*, medial; *Lmax*, maximum length; *Lmin*, distance between the sustentaculum tali and the tuber calcanei; *tuber*, tuber calcanei. All measurements given in millimetres

**Table 2:** Measurements of dental and post-cranial elements of *Samotherium major* from Kemiklitepe-E.

**Element**	**Inventory number**	**Sin/Dex**	**LD3**	**ID3**	**LD4**	**ID4**		
Maxilla	UEK-E/PV-4549	dex	31.62	22.83	33.27	28.38		
**Element**	**Inventory number**	**Sin/Dex**	**TDdis**	**APDdis**	**TDdis-tuber**	**APDdis-tuber**		
Metacarpus	UEK-E/PV-4529	sin	100.42	54.61	96.22	44.79		
**Element**	**Inventory number**	**Sin/Dex**	**Lmax**	**Lmin**	**TDmax**	**APDmax**	**TDtuber**	**APDtuber**
Calcaneus	UEK-E/PV-4531	dex	202.8	130.31	61.35	90.36	59.33	60.32
**Element**	**Inventory number**	**Sin/Dex**	Llat	**Lmed**	**TDprox**	**TDdis**		
Astragalus	UEK-E/PV-4499	sin	-	88.35	-	-		
Astragalus	UEK-E/PV-4535	sin	-	93.64	-	72.65		
**Element**	**Inventory number**	**Sin/Dex**	**TD**	**APDmed**	**H1**	**H2**		
Naviculo-cuboideum	UEK-E/PV-4537	dex	91.90	71.99	24.24	40.27		
Naviculo-cuboideum	UEK-E/PV-4538	sin	92.53	75.49	27.12	40.61		
Naviculo-cuboideum	UEK-E/PV-4539	dex	91.15	72.05	23.96	40.00		
**Element**	**Inventory number**	**Sin/Dex**	**TDdisart**	**APDdisart**	**TDdis-tuber**	**APDdis-tuber**		
Metatarsus	UEK-E/PV-4528	sin	92.71	51.90	92.56	46.18		
Metatarsus	UEK-E/PV-4530	dex	92.92	52.90	92.39	-		
**Element**	**Inventory number**	**L**	**TDprox**	**APDprox**	**TDdis**	**APDdis**		
Proximal phalanx	UEK-E/PV-4540	102.54	49.85	52.47	43.03	32.97		
Proximal phalanx	UEK-E/PV-4543	-	47.72	52.66	-	-		
Middle phalanx	UEK-E/PV-4545	59.05	43.26	-	37.85	-		
Middle phalanx	UEK-E/PV-4547	53.61	44.53	42.59	42.67	41.63		
Middle phalanx	UEK-E/PV-4548	55.16	40.86	48.01	35.55	40.98		

*Sin*, left; *Dex*, right; *L*, length; *I*, width; *D3*, upper third deciduous premolar; *D4*, upper fourth deciduous premolar; *APD*, antero-posterior diameter; *TD*, transverse diameter; *prox*, proximal; *dis*, distal; *med*, medial; *Lmax*, maximum length; *Lmin*, distance between the sustentaculum tali and the tuber calcanei; *tuber*, tuber calcanei; *H1*, height of the cubonavic ular on the level of the medial astragalar surface; *H2*, height of the cubonavicular on the level of the lateral astragalar surface; *dis-tuber*, distal tuberosity on the head of the metatars us; *art*, articular condyle. All measurements given in millimetres

### Institutional and anatomical abbreviations

D3: upper third deciduous premolar; D4: upper fourth deciduous premolar; EUNHM: Natural History Museum of Ege University; KTA: Kemiklitepe-A; KTB: Kemiklitepe-B; KTC: Kemiklitepe-C; KTD: Kemiklitepe-D; UEK-E: Uşak-Eşme-Kemiklitepe-E; MTA: Geological Survey of Turkey; PV: Palaeontology-Vertebrate.

## Systematic palaeontology

Class Mammalia Linnaeus, 1758

Order Cetartiodactyla Montgelard, Catzeflis and Douzery, 1997

Family Giraffidae Gray, 1821

Subfamily Sivatheriinae Zittel, 1893

Genus *Helladotherium* Gaudry, 1860

*Helladotherium duvernoyi* Gaudry and Lartet, 1856

### Material:

**Fig. 2:**
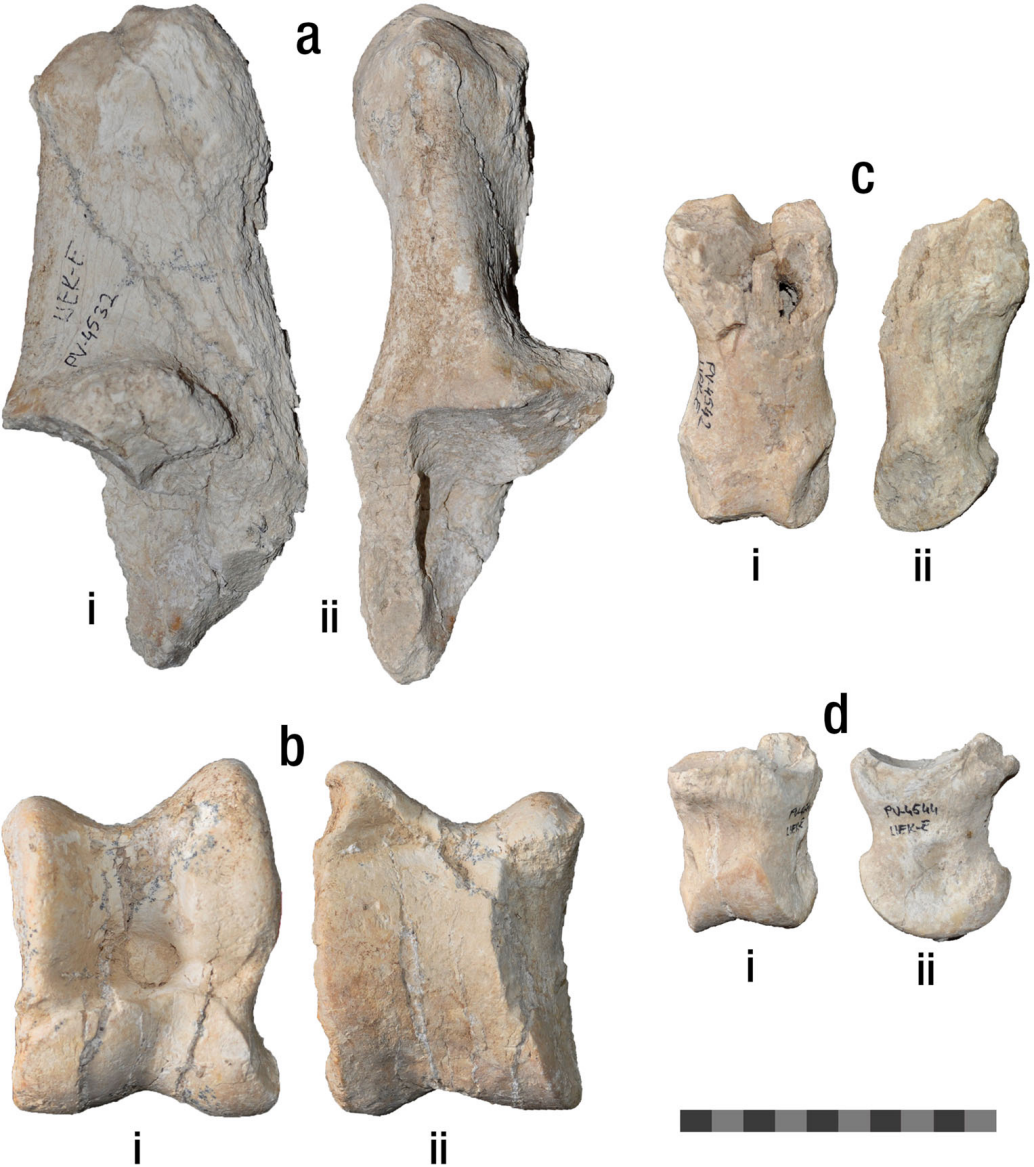
Postcranial material of *Helladotherium duvernoyi* from Kemiklitepe-E. **a** UEK-E/PV-4532, right calcaneus, (i) medial and (ii) dorsal view. **b** UEK-E/PV-4534, left astragalus, (i) dorsal and (ii) plantar view. **c** UEK-E/PV-4542 proximal phalanx, (i) dorsal and (ii) medial view. **d** UEK-E/PV-4544, middle phalanx, (i) dorsal and (ii) medial view. Scale equals 10 cm

(Fig. 2; Table 1) UEK-E/PV-4532, right calcaneus; UEK-E/PV-4533, left calcaneus; UEK-E/PV-4534, left astragalus; UEK-E/PV-4536, right astragalus (fragmented); UEK-E/PV-4541, proximal phalanx; UEK-E/PV- 4542, proximal phalanx; UEK-E/PV-4544, middle phalanx; UEK-E/PV-4546, middle phalanx

### Descriptions:

Calcanei are represented by two specimens (UEK-E/PV-4533 and UEK-E/PV-4532) with the latter missing the malleolar facet (Fig. 2a) and the former missing the distal part. In both specimens, the medial and lateral surfaces of the body, as well as the dorsal and plantar edges are parallel to each other. The tuber calcanei is well developed and protruding slightly more laterally. The depression for the tendon of the superficial digital flexor muscle is shallow and relatively narrow. The articular surface for the astragalus is concave with a weak crest running dorsoventrally. The sustentaculum tali is slightly narrow. The articular surface for the naviculocuboideum bone is narrow and strongly bent distally.

UEK-E/PV-4534 represents a complete astragalus (Fig. 2b). In dorsal aspect, the median ridge of the trochlea is thinner than the lateral one, with the latter being notably curved. The central fossa is oval-shaped, well-defined and shallow. The medial surface of the head is more developed than the lateral surface. The latter is slightly curved, with the lip extending laterally. In plantar view, the intratrochlear notch is deep, and the proximal triangular fossa is shallow and well limited by a medial and a lateral crest proximally, as well as the extension of the interarticular groove distally. The medial ridge is positioned close to the intratrochlear groove and a weak medial scala can be seen towards the head. The distal intracephalic fossa is not well defined and almost absent. UEK-E/PV-4536 represents a heavily fragmented astragalus with only the medial part preserved. Even though more than half of the astragalus is missing, in dorsal aspect, one can observe the very strong and round medial bulge at the collum tali, which can also be observed in UEK-E/PV-4534. In plantar view, the ventral articular surface shows a weak but well-defined medial scala, while distally, there is no sign of an intracephalic fossa.

The proximal phalanges (UEK-E/PV-4541 and UEK-E/PV-4542) are robust, with the base and head extending very insignificantly mediolaterally (Fig. 2c). On the proximal fovea articularis, the lateral articular facet is twice as wide as the medial articular surface, with the groove between them being wide and shallow. In plantart aspect, the plantar tubercles are robust and long.

The middle phalanges (UEK-E/PV-4544 and UEK-E/PV-4546) are rectangular in dorsal view (Fig. 2d). On the fovea articularis, the lateral articular surface is much longer dorsoventrally than the medial one, extending over the very strong postero-lateral tubercle. There is slight protuberance all around the base of the phalanx.

Subfamily Palaeotraginae Pilgrim, 1911

Genus *Samotherium* Forsyth-Major, 1888

*Samotherium major* Bohlin, 1926

### Material:

**Fig. 3:**
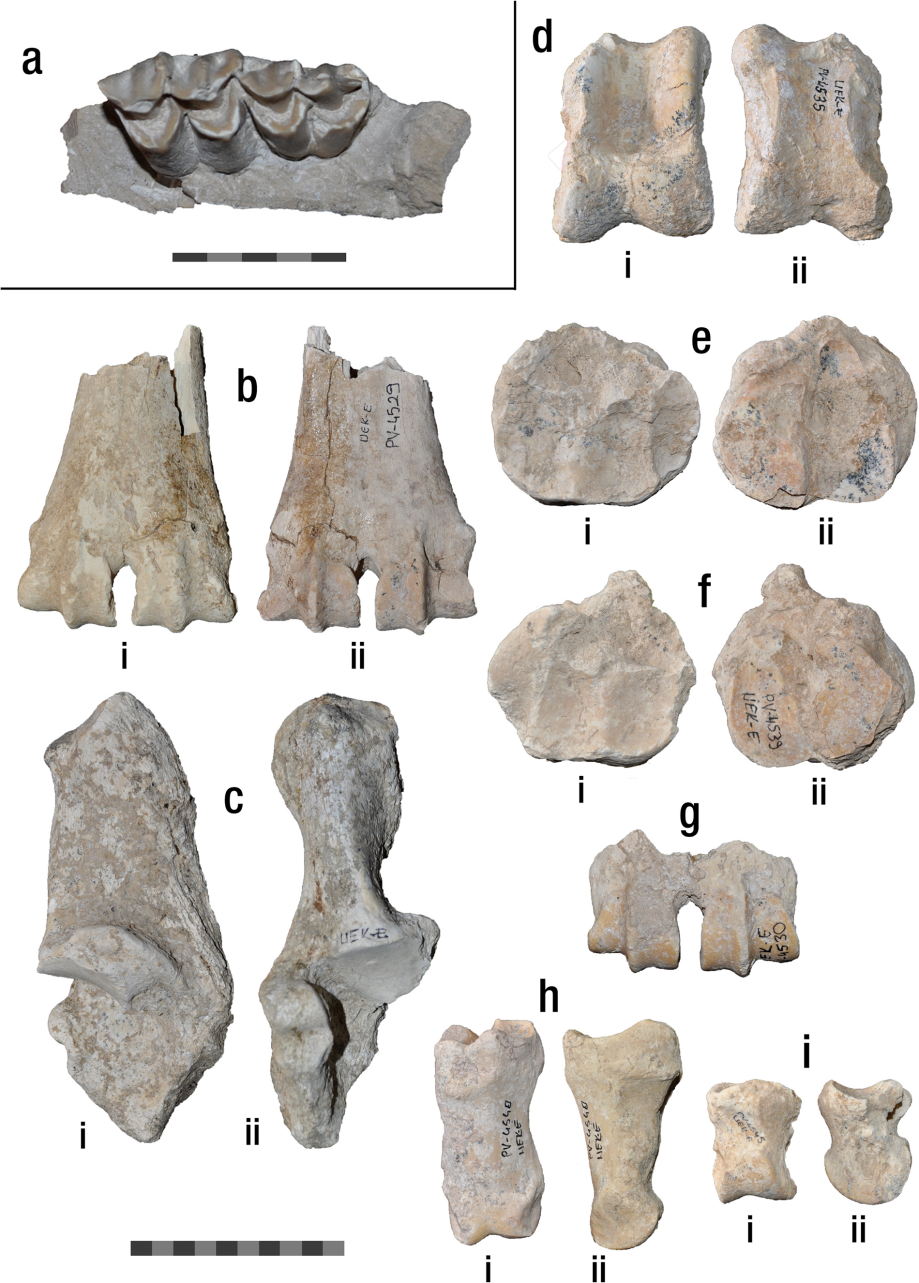
Fossil dental and post-cranial material of *Samotherium major* from Kemiklitepe-E. **a** UEK­E/PV-4549, right maxilla fragment with D3 and D4 (scale equals 5 cm). **b** UEK­E/PV-4529, distal part of left metacarpus, (i) dorsal and (ii) plantar aspect. **c** UEK-E/PV-4531, right calcaneus, (i) medial and (ii) dorsal aspect. **d** UEK-E/PV-4535, right astragalus, (i) dorsal and (ii) plantar aspect. **e** UEK­E/PV-4538, left naviculo-cuboideum, (i) proximal and (ii) distal aspect. **f** UEK­E/PV-4539, right naviculo-cuboideum, (i) proximal and (ii) distal aspect. **g** UEK­E/PV-4530, distal part of right metatarsus. **h** UEK­E/PV-4540, proximal phalanx, (i) dorsal and (ii) lateral aspect. **i** UEK­E/PV-4545, middle phalanx, (i) dorsal and (ii) lateral aspect. Scale equals 10 cm

(Fig. 3; Table 2) UEK-E/PV-4549, right maxilla fragment with D3 and D4; UEK-E/PV-4529, distal part of left metacarpus; UEK-E/PV-4531, right calcaneus; UEK-E/PV-4499, left astragalus (fragmented); UEK-E/PV-4535, right astragalus; UEK-E/PV-4537, right naviculo-cuboideum; UEK-E/PV-4538, left naviculo-cuboideum; UEK-E/PV-4539 right naviculo-cuboideum; UEK-E/PV-4528, distal part of left metatarsus; UEK-E/PV-4530, distal part of right metatarsus; UEK-E/PV-4540, proximal phalanx; UEK-E/PV-4543, proximal phalanx; UEK-E/PV-4545, middle phalanx; UEK-E/PV-4547, middle phalanx; UEK-E/PV-4548, middle phalanx.

### Description:

In UEK-E/PV-4549, only a small part of the maxilla is preserved (Fig. 3a). Therefore, no cranial characters can be described. Nevertheless, both D3 and D4 are complete, revealing the typical deciduous dental morphology of *S. major*. The D3 is molariform and trapezoid-shaped in occlusal aspect, with the anterior part being very rounded compared to the posterior one. The tip of the parastyle is broken but in labial aspect, there is no sign of bifurcation. The base of the parastyle is very strong, creating an approximately 55° angle with the axis of the paracone. The latter is well developed, and no labial cingulum is present. Lingually, the protocone is thin and simple without cingula or basal pillars. The anterior fossa is deep and crescent-shaped. A fold or spur is absent from the anterior lobe. The mesostyle is very strong, while the metacone and metastyle are very weak, with the latter projecting posterolabially. The metaconule is relatively robust and bearing a c-shaped metaconule fold. The D4 is completely molariform. The lingual cusps are very strong towards the crown and no cingula or styles are present. Labially, the mesostyle is very well developed. Parastyle and metastyle are slightly weaker but becoming progressively more robust towards the base of the crown. Both anterior and posterior fossa are deep and simple with no additional folds.

The metacarpus (Fig. 3b) is represented by an incomplete specimen (UEK-E/PV-4529), which lacks the proximal part completely. In palmar aspect, the central trough becomes very flat distally and there is a faint pyramidal rise. The keels of the distal condyles continue onto the shaft of the metacarpus.

The calcaneum (UEK-E/PV-4531) is complete with a biconcave body (Fig. 3c). The tuber calcanei is very well developed and slightly more robust laterally. A wide depression for the tendon of the superficial digital flexor muscle can be observed in plantar aspect. The sustentaculum tali is very strong. The articular surface for the astragalus is concave with a short crest running through it, dividing the surface in two parts. The surface for the naviculocuboideum bone is wide and slightly curved laterally. In dorsal aspect, the malleolar facet is positioned at the level of the coracoid process, and it is well developed with a wide concave groove right beneath it.

The astragalus (UEK-E/PV-4535; Fig. 3d), is slightly fragmented and the tip of the lateral ridge of the trochlea is missing. In dorsal aspect, the median ridge of the trochlea is thinner than the lateral ridge. The central fossa is very wide and shallow, and the groove of the trochlea is flattened. The medial surface of the head is notably more developed than the lateral surface. In plantar aspect, the intratrochlear notch is wide. The proximal triangular fossa is not preserved. The interarticular groove is long and narrow. The ventral articular surface is rectangular and clockwise tilted, relative to the body of the astragalus. The medial scala is almost absent. The distal intracephalic fossa is shallow but well-defined and separated into a deeper lateral area and a shallower and more distally placed medial area. UEK-E/PV-4499 represents only the medial part of an astragalus. Even though more than half of the astragalus is missing one can notice that in the ventral articular surface, there is a very faint media scala, as well as a distinct concavity on the level of the distal intracephalic fossa.

The naviculocuboideum (UEK-E/PV-4537, UEK-E/PV-4538 and UEK-E/PV-4539) is almost round in proximal view (Fig. 3e–f). The lateral astragalar facet is slightly wider and longer than the medial one. The calcaneal facet is wide and not extending behind the lateral peak. In distal view, the facet for the metatarsal and the external cuneiform are semi-circular, with the latter being notably smaller. The medial cuneiform facet is round and isolated from the facet of the external cuneiform.

The metatarsus is represented by two incomplete specimens. UEK-E/PV-4528 maintains the distal part of the metatarsus with a small part of the diaphysis. In dorsal aspect, the dorsal longitudinal grove is wide, becoming wider towards the intertrochlear notch. In plantar view, the distal part of the central trough is flat and the keels of the distal condyles continue onto the diaphysis. UEK-E/PV-4530 preserves only the distal epiphysis with the distal condyles, representing a young individual (Fig. 3g).

The proximal phalanges (UEK-E/PV-4540 and UEK-E/PV-4543) are robust, with the base slightly extending mediolaterally (Fig. 3h). On the proximal fovea articularis, the lateral facet is slightly larger than the medial one and the groove between them is relatively deep. The palmar/plantar tubercles are strong but very short and limited on the proximal epiphysis.

The middle phalanges (UEK-E/PV-4545, UEK-E/PV-4547 and UEK-E/PV-4548) are rectangular in dorsal view and slightly compressed mediolaterally (Fig. 3i). On the proximal fovea articularis, the lateral and medial articular surfaces are almost equally developed. The postero-lateral tubercle is prominent but not significantly robust.

## Discussion

Although the giraffid remains from Kemiklitepe-E are scarce, the available fossils reveal the presence of two classic Turolian large giraffids: *Helladotherium duvernoyi* and *Samotherium major*. Based on the above described material, the morphological comparison of the two taxa is demonstrated below.

**Fig. 4:**
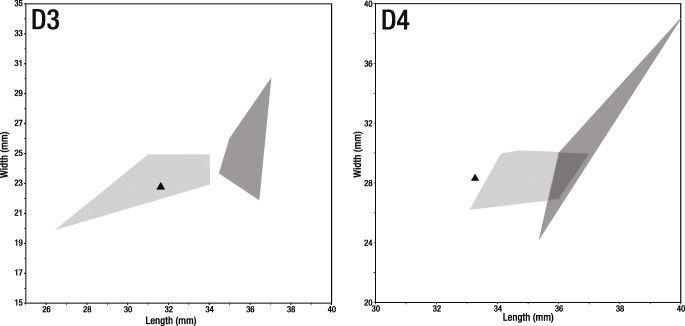
Scatter plots exhibiting the size of deciduous dentition of *Helladotherium duvernoyi* and *Samotherium major*. Light grey areas represent the morphospaces of *Samotherium major*. Dark grey areas represent the morphospaces of *Helladotherium duvernoyi*. Black triangles represent the specimens of *S. major* from Kemiklitepe-E. Measurements given in millimetres (data from Kostopoulos and Koufos 2006; Kostopoulos 2009)

The deciduous dentition of *Helladotherium duvernoyi* is always larger than that of *Samotherium major* (Fig. 4). Morphologically, the anterior lobe of the D3 is much longer in *H. duvernoyi* than in *S. major*, and the parastyle is bifurcated (Kostopoulos and Koufos 2006; Kostopoulos 2009). Labial and lingual cingula, well developed folds and spurs are plesiomorphic features characterising *Helladotherium* and all other Sivatheriinae (Colbert 1935; Geraads 1974; Hamilton 1978; Geraads and Güleç 1999; Kostopoulos and Koufos 2006; Kostopoulos 2009). On the contrary, deciduous dentition of *Samotherium*, and palaeotragines in general, shows a simpler morphology. The anterior lobe of the D3 is fully molariform. Additionally, the parastyle is not bifurcated and the D4 is also fully molarised. Both D3 and D4 are missing strong labial and lingual cingula.

The metacarpals of *H. duvernoyi* are generally stronger and bigger than the metacarpals of *S. major* (Kostopoulos 2009). Additionally, the protruding keels of the distal condyles onto the distal part of the diaphysis is a characteristic feature of *S. major*, which also applies to the metatarsals (Ríos et al. 2016).

**Fig. 5:**
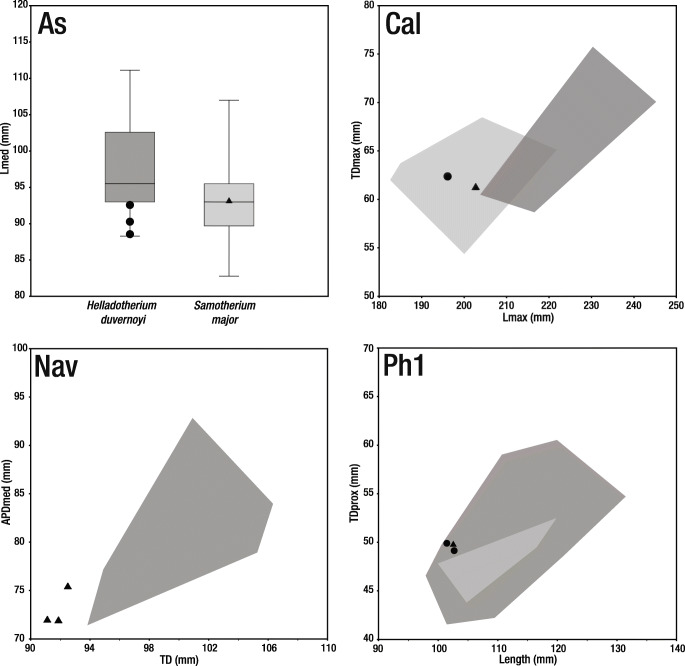
Dispersion plots showing the size of tarsal bones and proximal phalanges of *Helladotherium duvernoyi* and *Samotherium major* from Kemiklitepe-E. Light grey areas represent the *S. major* morphospaces. Dark grey areas represent the *H. duvernoyi* morphospaces. Black circles and triangles represent the *H. duvernoyi* and *S. major* specimens from Kemiklitepe-E, respectively. In the boxplot, each box represents 50% of the range of the premolar length, while the top and the bottom bars represent the overall range of the length. Horizontal line in the centre of each box represents the median of the sample. All measurements given in millimetres (data from Geraads 1994; Iliopoulos 2003; Kostopoulos and Saraç 2005; Kostopoulos and Koufos 2006; Kostopoulos 2009; Xafis et al. 2019b)

The astragali and calcanei of *H. duvernoyi* and *S. major* exhibit a large metrical overlapping (Fig. 5). Nevertheless, a number of morphological features can be used to separate the two taxa. In the astragali of *Samotherium*, the lateral and medial ridges of the trochlea are parallel to each other; the intratrochlear notch is shallow and the distal intracephalic fossa is deep and very well defined (Kostopoulos 2009; Solounias and Danowitz 2016a). On the contrary, the astragali of *Helladotherium* exhibit non-parallel trochlear ridges, a deeper intratrochlear noch and a very faint to absent distal intracephalic fossa (Kostopoulos 2009; Solounias and Danowitz 2016a). The calcanei of *Samotherium* show a deep depression for the superficial digital flexor muscle; the surface for the naviculocuboideum is very wide, and the malleolar facet is positioned on the level of the coracoid process. Respectively, on the calcanei of *H. duvernoyi,* the depression for the superficial digital flexor muscle is shallow, and the surface for the naviculocuboideum is narrow and strongly bent distally.

Even though the naviculocuboideum of *Helladotherium duvernoyi* exhibits a large metrical variation, the Kemiklitepe-E specimens are notably smaller, falling out of the *Helladotherium* morphospace (Fig. 5). On the naviculocuboideum of *S. major*, the medial crest is much weaker and the astragalar facets are shorter than in *H. duvernoyi*. Additionally, the specimens found at Kemiklitepe-E display a calcanear facet, which is short and not extending behind the lateral crest. This is an uncommon character for sivatheriines and a diagnostic trait for samotheriines (Kostopoulos 2009; Nishioka et al. 2014; Solounias and Danowitz 2016a).

The phalanges of *H. duvernoyi* and *S. major* are very similar morphologically, as well as metrically (Fig. 5). The proximal phalanx of *H. duvernoyi* is longer and more robust than those of *S. major*. Additionally, the palmar/plantar tuberosities of the proximal epiphysis are strong and long, running through almost half of the phalanx’s body (Kostopoulos 2009). On the contrary, in *S. major*, the proximal phalanx is slightly narrow medio-laterally, and has very short palmar/plantar tubercles. The middle phalanges of *H. duvernoyi* are also larger, bearing a very strong postero-lateral tubercle, which is notably smaller in *S. major* (Kostopoulos 2009).

*Helladotherium* is a monospecific genus primarily known from various Greek sites, with Pikermi being the type locality of the taxon (Gaudry 1861). *Helladotherium duvernoyi* is one of the most abundant late Miocene giraffids of the Greco-Iranian Province, with a wide chronostratigraphic range from late Vallesian to late Turolian (Kostopoulos et al. 1996; Kostopoulos and Koufos 2006; Kostopoulos 2009; Koufos et al. Koufos et al. 2009). In Anatolia, the taxon has been reported from Amasya, Eski Bayırköy, Eskihisar Mine, Kavakdere, Mahmutgazi, Yukarısazak, Bala Yaylaköz, Duzyayla, Gülpınar and Akkaşdağı (Becker-Platen et al. 1975; Tuna 1999; Kostopoulos and Saraç 2005; the NOW community 2019).

*Samotherium major* is a typical middle Turolian (MN12) taxon, which replaced the similar but smaller *Samotherium boissieri* Forsyth-Major (1888) at about 7.4 million years ago, at the transition of lower to middle Turolian (Kostopoulos et al. 2003; Kostopoulos 2009). The type locality of *S. major* is Samos (Forsyth-Major 1888; Bohlin 1926; Kostopoulos 2009), but it has also been recorded from Vathylakkos (Geraads 1978), Kerasia (Iliopoulos 2003), Kemiklitepe A/B (Geraads 1994), Akkaşdağı (Kostopoulos and Saraç 2005), Mahmutgazi (Geraads 2017), Taşkinpaşa (Şenyürek 1954) and Thermopigi (Xafis et al. 2019b). Recently, *S. major* was also described from Maragheh, by two postcranial elements (Solounias and Danowitz 2016b), representing the easternmost occurrence of the taxon. The more frequent presence of *S. major* in Anatolia is particularly important for verifying and depicting the Eastern origin of this rare taxon and its western migration during the Turolian.

In a previous taxonomic study on giraffid remains from Kemiklitepe, Geraads (1994) described *Samotherium major* and *Palaeotragus rouenii* from the upper horizons (KTA, KTB and KTC) and *P. rouenii* and *Samotherium*? sp. from the lower horizon (KTD). Therefore, the presence of *Helladotherium* in UEK-E constitutes the first record of this taxon from Kemiklitepe. Kostopoulos (2009) suggested that the absence of *Helladotherium* from KTA and KTB indicated a later arrival of this taxon into the Eastern Aegean area. The new findings presented herein show that *Helladotherium* was in fact already present in the area. However, the scarcity of material from that time period, not only at UEK-E, but in Central and Western Anatolia (e.g. Kemiklitepe A/B, Akkaşdağı, Mahmutgazi, Şerefköy-2) in general, agrees with the assumption that large sivatheriines only just migrated into the area during the middle Turolian and then subsequently became more dominant (Geraads 1994; Kostopoulos and Saraç 2005; Kaya et al. 2012; Geraads 2017).

*Samotherium major* is the most common taxon at Kemiklitepe. *Palaeotragus rouenii* seems to be a consistent taxon for the lower and higher stratigraphic horizons, even though it was not found at UEK-E. Geraads (1994) also described *Samotherium*? sp. from KTD as a giraffid of intermediate size between *S. boissieri* and *S. major*. In this study, the majority of the *Samotherium*? sp. specimens are not depicted, but are all described in detail. Geraads noted the similarity of some skeletal elements with those of *Samotherium* (*Alcicephalus*) *neumayri* Rodler and Weithofer (1890), and also mentioned the clear presence of distal intracephalic fossa on the astragalus. The latter constitutes a distinct character for samotheriines and is present in both *S. major* and *S. neumayri* (Solounias and Danowitz 2016a; Xafis et al. 2019a). *Samotherium (Alcicephalus) neumayri* is one of the most common late Miocene giraffid taxa at the eastern boundary of the Pikermian Biome, and it is reported from its type locality, Maragheh, as well as from North China (de Mecquenem 1924-1925; Gaziry 1987; Solounias and Danowitz 2016b; Rodler and Weithofer 1890). A recent study on fossil giraffids from Kavakdere revealed the presence of *S. neumayri* in the early Turolian (MN11) of Anatolia (Xafis et al. 2019a). Considering the age of KTD, as well as the size and characters of what was described as *Samotherium*? sp. by Geraads (1994), it is suggested that the specimens most likely represent *S. neumayri*.

*Helladotherium duvernoyi* and *Samotherium major* have very similar body masses and sizes, justifying that they could reach the same vegetation heights (Merceron et al. 2018). Previous work has shown that even though *H. duvernoyi* bears some mixed-feeding features (Solounias and Dawson-Saunders 1988; Solounias et al. 1999), it is generally accepted that the taxon was a large browsing sivatheriine (Solounias et al. 2000; Solounias et al. 2010; Solounias et al. 2013; Solounias and Danowitz 2016a). *Samotherium major* was primarily considered a grazer (Solounias and Moelleken 1993; Solounias et al. 1988, 1999, 2000). However, recent studies show that *S. major* was more adapted to mixed-feeding dietary habits (Solounias et al. 2010; Solounias et al. 2013). The latest comprehensive study on the diet of extinct giraffids, using dental microwear textural analysis, showed that *H. duvernoyi* was indeed a leaf browser, while *S. major* is considered a mixed-feeder, even though the microwear signal of the two large herbivores is very similar (Merceron et al. 2018). This, suggests that the two giraffid taxa coexisted in the large mammal community at Kemiklitepe-E through resource partitioning, which drove *H. duvernoyi* and *S. major* towards different food options.

**Fig. 6:**
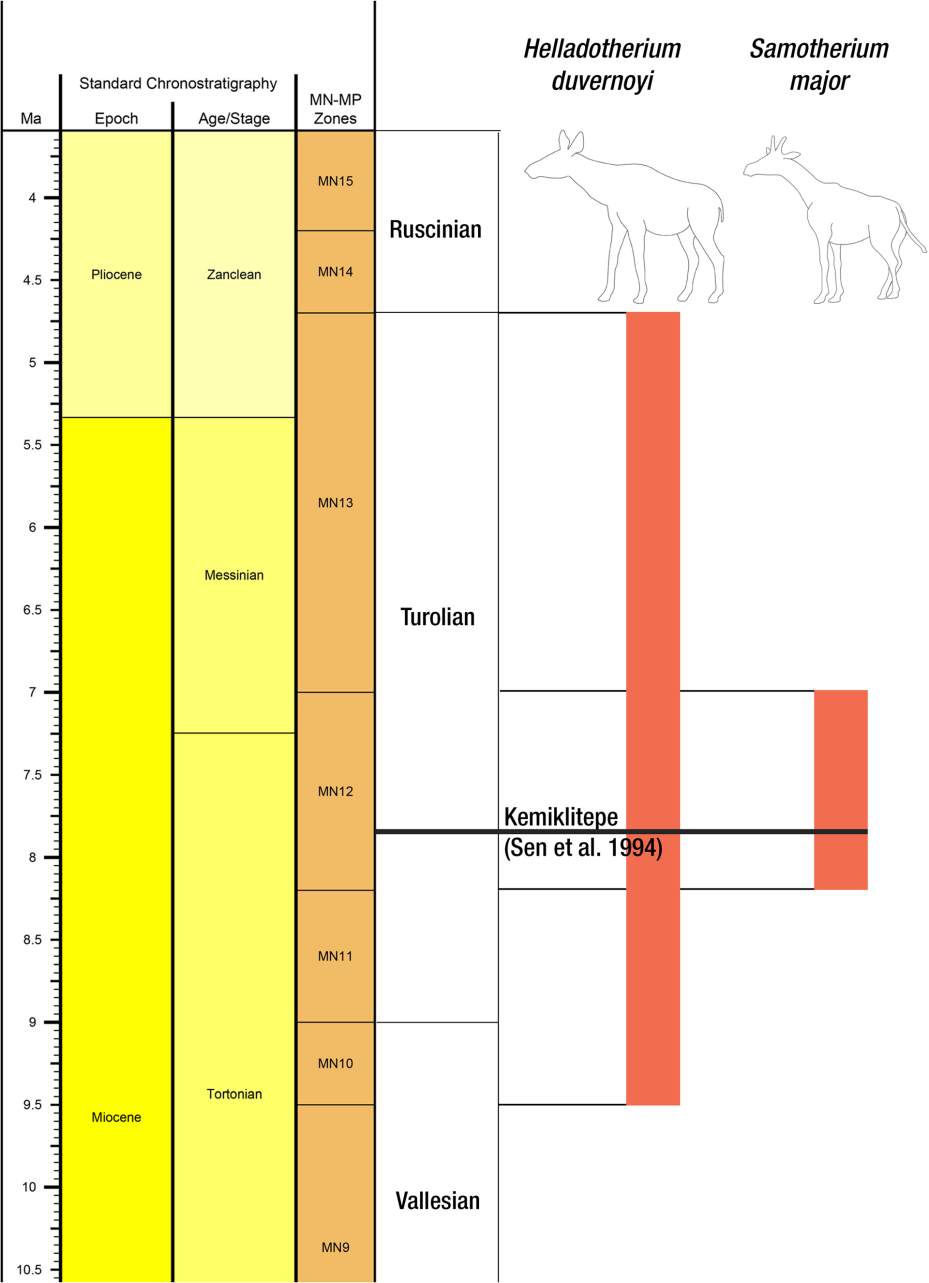
Stratigraphic distribution of the *Helladotherium duvernoyi* and *Samotherium major*. Dark line indicates the stratigraphic position of Kemiklitepe, as proposed by Set et al. (1994).

*Helladotherium duvernoyi* has a wide chronostratigraphic range from late Vallesian to late Turolian, while *Samotherium major* only appears in middle Turolian localities (Kostopoulos et al. 1996; Kostopoulos and Koufos 2006; Kostopoulos 2009; Koufos et al. 2009; Fig. 6). The two taxa coexist in Samos, Kerasia, Akkaşdağı and Thermopigi (Forsyth-Mayor 1888; Bohlin 1926; Iliopoulos 2003; Kostopoulos and Saraç 2005; Kostopoulos 2009; Xafis et al. 2019b), consistently occurring in middle Turolian sediments. Also, preliminary determination of other mammalian remains reveals a classic Turolian fauna. Conclusively, a middle Turolian age (MN12) is suggested for Kemiklitepe-E, which is in agreement with the synchronous KTA, KTB and KTC of the classic Kemiklitepe locality (Sen et al. 1994).

## Conclusions

A new fossiliferous locality named Kemiklitepe-E is presented for the first time, located approximately 350 m NW of the classic Kemiklitepe site. The newly discovered outcrop has yielded a descent amount of large mammalian fossil remains including representatives of Proboscidea, Equidae, Chalicotheriidae, Bovidae and Giraffidae. The fossil giraffid remains are comprehensively described herein and exhibit the presence of two large taxa: *Helladotherium duvernoyi* and *Samotherium major*. The latter constitutes the most common giraffid occurring at Kemiklitepe, while *H. duvernoyi* is reported for the first time. In addition, it is also suggested that the specimens assigned to *Samotherium*? sp. by Geraads (1994) from the stratigraphically lower KTD horizon, probably represent *S. neumayri*. A recent comprehensive dental wear analysis of the family Giraffidae showed that *H. duvernoyi* and *S. major* exhibit almost identical microwear signals. However, *S. major* is considered to be more of a generalist and therefore, the two taxa successfully coexisted at Kemiklitepe-E through niche partitioning. The coexistence of *H. duvernoyi* and *S. major* has been previously reported from Samos (Forsyth-Major 1888; Bohlin 1926; Kostopoulos 2009), Kerasia (Iliopoulos 2003), Akkaşdağı (Kostopoulos and Saraç 2005), and Thermopigi (Xafis et al. 2019b), and is indicative of middle Turolian (MN12) age.
